# Medical College of Ohio

**Published:** 1834

**Authors:** 


### IX. Medical College of Ohio.

Our own school has (for it) the respectable number, of 83 pay pupils, and 8 beneficiaries; and but for that "scourge of nations" and medical colleges the *cholera*, it would have been still larger. It is perhaps difficult to account on this principle for so great a falling off in the number its charity scholars. When the power of the State was exercised, in favour of this important department of the college, it was much fuller; and it is worther of consideration, whether the legislature, should not by law, offer a premium out of the state treasury, to such indigent students, as are unwilling to *accept* of the tickets of the professors. Such a measure might be calculated to correct the pernicious effects which the cholera has exerted upon our otherwise flourishing institution. As the state have given 35 or 36 thousand dollars to the college, it can scarcely be necessary to appropriate much more; and the remainder of what is intended for the support of medical education, might advantageously be employed in procuring the attendance of pupils.

